# Systematic Review and Meta-Analysis of *SERPINE1* 4G/5G Insertion/Deletion Variant With Circulating Lipid Levels

**DOI:** 10.3389/fcvm.2022.859979

**Published:** 2022-06-23

**Authors:** Zhi Luo, Yang Liu, Hang Li, Yawen Zhou, Yuanyuan Peng, Xuan Lin, Ying Fang, Jing Wan, Baozhu Wei

**Affiliations:** ^1^Department of Cardiology, Zhongnan Hospital of Wuhan University, Wuhan University, Wuhan, China; ^2^Department of Endocrinology, China Resources and WISCO General Hospital, Wuhan, China; ^3^Department of Geratology, Zhongnan Hospital of Wuhan University, Wuhan University, Wuhan, China; ^4^Institute of Myocardial Injury and Repair, Wuhan University, Wuhan, China

**Keywords:** rs1799889, polymorphism, lipid, coronary artery disease, Asian

## Abstract

**Background:**

Recent studies have shown that the 4G/5G insertion/deletion variant of *SERPINE1* (rs1799889) is closely linked to coronary artery disease (CAD). This study aims to clarify the effects of the rs1799889 variant on lipid levels and to insight into the mechanisms underlying the rs1799889 variant and CAD.

**Methods and Results:**

By searching PubMed and the Cochrane databases for studies published before 31 October 2021, 40 studies conducted on a total of 13,117 subjects were included for the analysis. The consistent findings for the effects of the 5G allele of rs1799889 variant on lipid metabolism were the significantly decreased triglycerides (TG) [standardized mean difference (SMD) = –0.12, 95% CI = –0.21 to 0.03, *P* = 0.01], total cholesterol (TC) (SMD = –0.12, 95% CI = –0.17 to 0.06, *P* < 0.001), and low-density lipoprotein cholesterol (LDL-C) (SMD = –0.13, 95% CI = –0.23 to 0.03, *P* = 0.01) levels. Intriguingly, the significant effects of the rs1799889 variant on LDL-C (SMD = –0.15, 95% CI = –0.26 to 0.05, *P* < 0.01) and TC (SMD = –0.17, 95% CI = –0.27 to 0.07, *P* < 0.01) levels were primarily observed in the Asian population. However, the significant effect of the rs1799889 variant on high-density lipoprotein cholesterol (HDL-C) (SMD = 0.26, 95% CI = 0.03–0.48, *P* = 0.03) levels was detected only in female subjects.

**Conclusion:**

The rs1799889 variant of *SERPINE1* is a protective genetic factor against CAD, the Asian population with the 5G allele of the rs1799889 variant may have a reduced CAD risk.

## Introduction

The plasminogen activator inhibitor-1 (*PAI-1*), also known as *SERPINE1*, is a member of the serine protease inhibitor superfamily and plays a major role in regulating fibrinolysis by inhibiting plasminogen activity.

The *SERPINE1* gene is located on the long arm of human chromosome 7 (7q21.3-22). The most-studied rs1799889 variant, also known as 4G/5G insertion/deletion sequence, is located in the *SERPINE1* promoter region and formed by the 5th guanine (G base) insertion or deletion in the 4G sequence at position-675th base. The 5G allele has lower transcriptional activity than the 4G allele (1) and subjects with homozygous 5G allele have about 25% lower circulating PAI-1 concentrations than subject’s possession of homozygous 4G allele (2).

Notably, a set of elegant experiments showed that SERPINE1 may affect lipid metabolism. For instance, *SERPINE1* knockout (*SERPINE1*–*^/^*–) inhibits the expression of adipogenic genes (3) and ameliorates dyslipidemia (4) in mice. Consistently, transplanting white adipose tissue from *SERPINE1*–*^/^*– mice into obese mice reduces plasma triglycerides (TG) and total cholesterol (TC) levels (5). Moreover, inhibition of *SERPINE1* expression decreases proprotein convertase subtilisin/kexin type 9 (PCSK9) and increases low-density lipoprotein receptor (LDLR) levels (6). Taken together, these indicate that the expression levels of SERPINE1 are closely linked to lipid metabolism. Therefore, the 5G allele of the rs1799889 variant may affect lipid levels due to its profound impact on SERPINE1 expression levels (1, 2).

Currently, a series of meta-analyses (7–10) showed that the 5G allele of the rs1799889 variant largely decreased coronary artery disease (CAD) risk; however, the specific mechanism is unknown. Therefore, this study is conducted to investigate the effects of the rs1799889 variant on lipid levels and to clarify the mechanistic basis for the correlations between the rs1799889 variant and CAD.

## Materials and Methods

The study design followed the Preferred Reporting Items for Systematic Reviews and Meta-Analyses (PRISMA) (11).

### Literature Search

The literature search was carried out by using PubMed, Medline, Embase, Cochrane Library, Web of Science, Google Scholar, Foreign Medical Journal Service, and Excerpta Medica for manuscripts published before 31 October 2021, by entering the following keywords: (“SERPINE1,” “PAI-1,” or “plasminogen activator inhibitor-1”), (“4G/5G,” “insertion/deletion,” or “rs1799889”), (“variant,” “mutant,” or “polymorphism”), and (“lipid,” “lipids,” “lipid metabolism,” “lipoprotein,” “cholesterol,” “blood lipid,” “serum lipid,” or “circulating lipid”).

### Inclusion Criteria and Exclusion Criteria

The inclusion criteria of this meta-analysis were as follows. (1) The studies investigated the effects of the rs1799889 variant on lipid levels. (2) The studies at least provided one of the four lipid parameters (TG, TC, LDL-C, and HDL-C). (3) The studies provided the genotype frequencies of the rs1799889 variant. (4) The rs1799889 variant offered the mean lipid levels with standard deviation (SD) or standard error (SE) by genotypes. (5) The interventional studies provided pre-intervention data. (6) The language of eligible studies was restricted to English or Chinese. The exclusion criteria of this meta-analysis include (1) studies that were not related to the rs1799889 variant, (2) studies that were not related to lipid levels, (3) studies not presenting genotype or allele counts, (4) studies having invalid data, (5) studies having incomplete data, (6) pedigree studies (7), overlapping studies, and (8) abstract, review, case report, meta-analysis, and animal studies.

### Subgroup Analysis

Subgroup analysis was carried out by ethnicity, gender, and health status. The ethnicity was divided into Caucasian, Asian, Turkish, and other ethnics. The healthy status was divided into patients with CAD, patients with type 2 diabetes mellitus (T2DM), and healthy subjects.

### Other Items

Refer to the previous publication (12) for more details about data extraction, data analysis, heterogeneity processing, and publication bias tests.

## Results

### Study Selection

**FIGURE 1:**
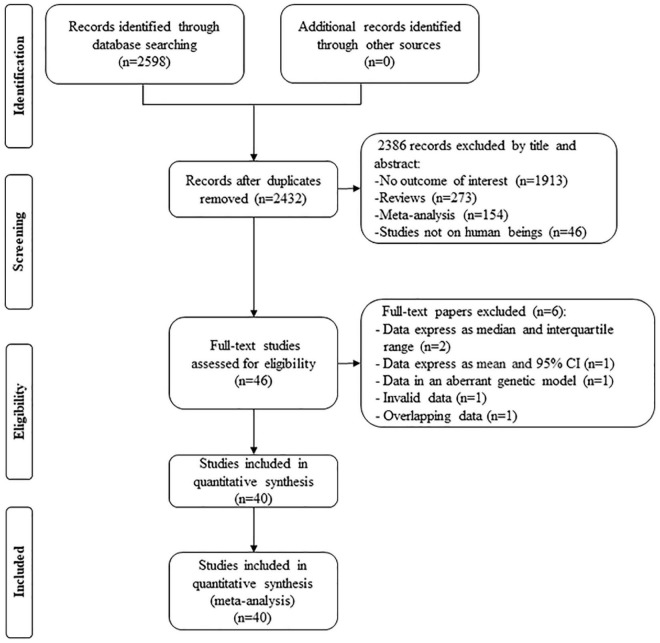
Flow diagram of the articles selection process.

By searching the PubMed and Cochrane database, 2,598 studies were screened; after excluding duplicates, 2,386 studies were removed by their title and abstract. Then, 46 full-text studies were included for assessing eligibility, in which two studies (13, 14) provided lipid data by the genotype of rs1799889 but expressed as a median and interquartile range, 1 study (15) provided lipid data by the genotype of rs1799889 but expressed as mean and 95% CI, 1 study (16) provided lipid levels by the genotype of rs1799889 but in an aberrant genetic model [(4G5G + 4G4G) vs. 5G5G], 1 study (17) provided invalid data, and 1 study (18) had subjects overlapping with other publications (19). Therefore, six studies were further excluded. Finally, 40 studies (13,117 subjects) were eligible for the analysis (Figure 1).

The characteristics of the eligible studies were summarized in Supplementary Table 1. The circulating lipid levels by the genotype of the *SERPINE1* rs1799889 variant were presented in Supplementary Table 2.

### Effects of the rs1799889 Variant on Circulating Lipid Levels

**FIGURE 2:**
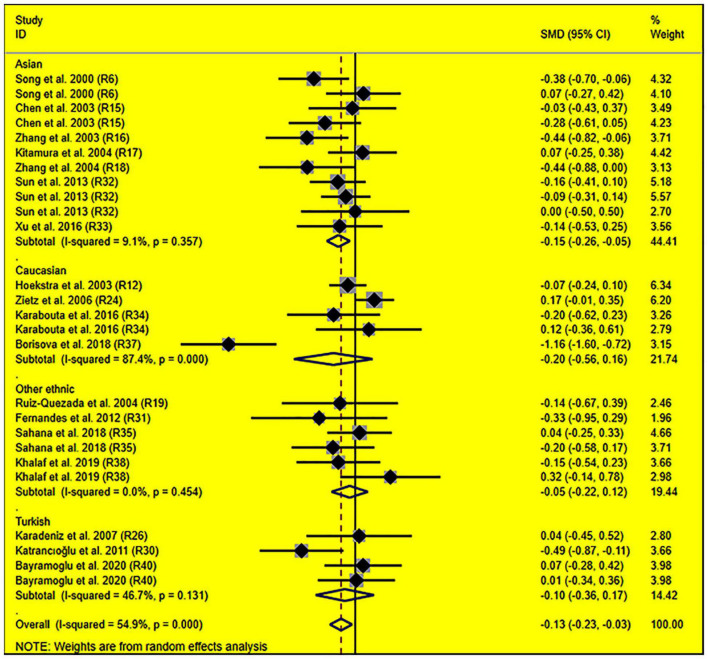
Forest plot of the meta-analysis between *SERPINE1* rs1799889 polymorphism and circulating LDL-C levels.

**FIGURE 3:**
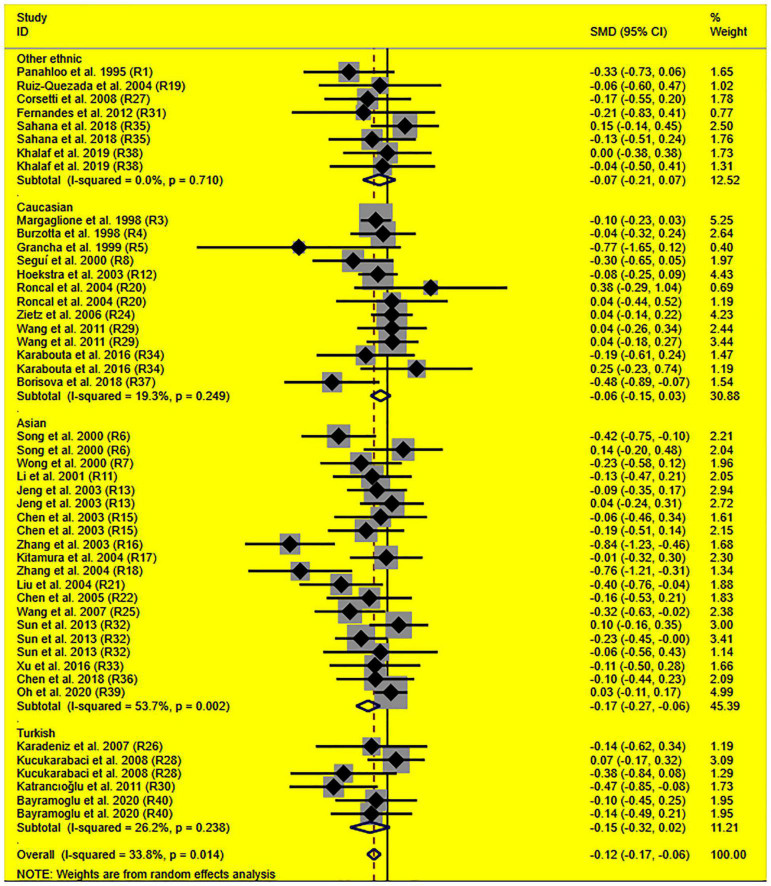
Forest plot of the meta-analysis between *SERPINE1* rs1799889 polymorphism and circulating TC levels.

**FIGURE 4:**
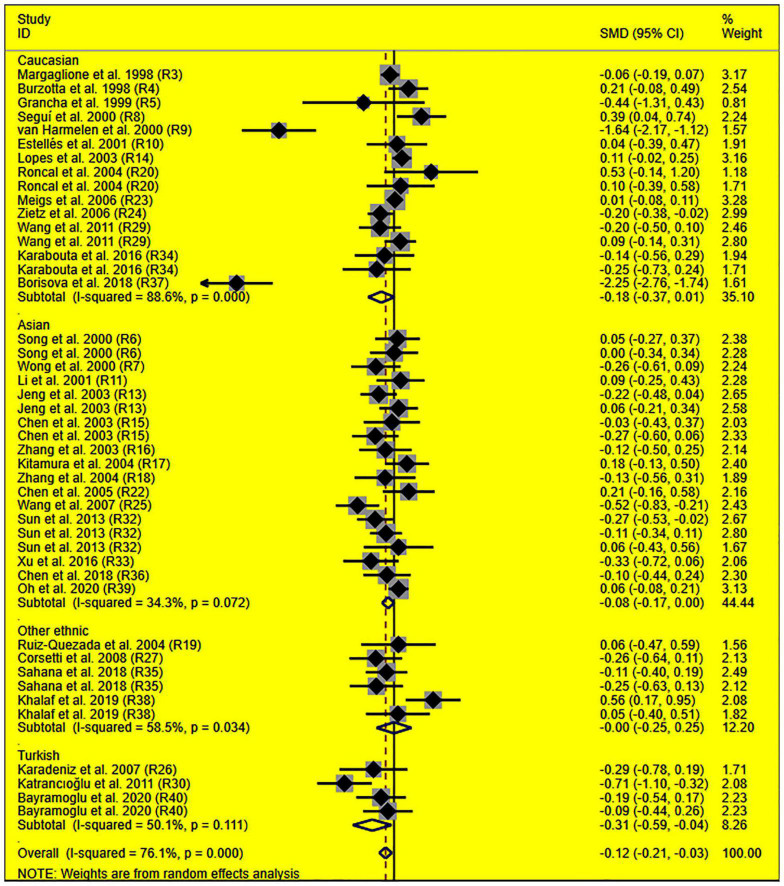
Forest plot of the meta-analysis between *SERPINE1* rs1799889 polymorphism and circulating TG levels.

The consistent findings for the effects of the rs1799889 variant on lipid metabolism were the significantly decreased LDL-C [standardized mean difference (SMD) = –0.13, 95% CI = –0.23 to 0.03, *P* = 0.01], TC [SMD = –0.12, 95% CI = –0.17 to 0.06, *P* < 0.001], and TG [SMD = –0.12, 95% CI = –0.21 to 0.03, *P* = 0.01] levels (Table 1 and Figures 2–4). After excluding the population that deviated from Hardy–Weinberg equilibrium (HWE), the significant effects of the rs1799889 variant on LDL-C, TC, and TG levels were also detected (Table 1).

**TABLE 1:** Meta-analysis of *SERPINE1* rs1799889 polymorphism with lipid levels.

Groups or subgroups	Comparisons (Subjects)	*P*_H_	SMD (95% CI)	*P*_SMD_
**TG**				
All	45 (11 597)	<0.001	−0.12 (−0.21 to −0.03)	0.01
Studies in HWE	33 (9 502)	<0.001	−0.11 (−0.22 to −0.00)	0.05
Caucasian	16 (6 551)	<0.001	−0.18 (−0.37 to 0.00)	0.06
Asian	19 (3 823)	0.07	−0.09 (−0.17 to 0.00)	0.06
Turkish	4 (458)	0.11	−0.31 (−0.59 to −0.04)	0.03
Other ethnic	6 (765)	0.03	−0.00 (−0.25 to 0.25)	0.99
Male	2 (461)	0.05	−0.03 (−0.42 to 0.37)	0.89
Female	6 (1 118)	0.47	−0.12 (−0.26 to 0.01)	0.07
CAD	5 (595)	0.80	−0.10 (−0.26 to 0.07)	0.25
T2DM	7 (2 472)	<0.01	0.01 (−0.17 to 0.19)	0.94
Healthy subjects	14 (4 819)	<0.001	−0.15 (−0.31 to 0.01)	0.07
**TC**				
All	47 (9 482)	0.01	−0.12 (−0.17 to −0.06)	<0.001
Studies in HWE	32 (6 425)	0.01	−0.13 (−0.20 to −0.05)	<0.01
Caucasian	13 (3 753)	0.25	−0.06 (−0.15 to 0.03)	0.18
Asian	20 (3 970)	<0.01	−0.17 (−0.27 to −0.07)	<0.01
Turkish	6 (791)	0.24	−0.15 (−0.32 to 0.02)	0.09
Other ethnic	8 (968)	0.71	−0.07 (−0.21 to 0.07)	0.33
Male	2 (461)	0.70	−0.06 (−0.26 to 0.14)	0.57
Female	7 (1 175)	0.34	−0.08 (−0.22 to 0.06)	0.27
CAD	5 (595)	0.01	−0.31 (−0.66 to 0.04)	0.08
T2DM	8 (1 698)	0.30	−0.09 (−0.20 to 0.03)	0.14
Healthy subjects	14 (3 270)	0.69	−0.09 (−0.17 to −0.02)	0.02
**LDL-C**				
All	26 (4 309)	<0.001	−0.13 (−0.23 to −0.03)	0.01
Studies in HWE	16 (2 226)	<0.001	−0.13 (−0.23 to −0.03)	0.02
Caucasian	5 (1 453)	<0.001	−0.20 (−0.56 to 0.16)	0.28
Asian	11 (1 737)	0.36	−0.15 (−0.26 to −0.05)	<0.01
Turkish	4 (458)	0.13 0.13	−0.10 (−0.36 to 0.17)	0.48
Other ethnicity	6 (661)	0.45	−0.05 (−0.22 to 0.12)	0.55
Female	5 (874)	0.88	−0.11 (−0.25 to 0.04)	0.16
CAD	4 (554)	0.08	−0.16 (−0.42 to 0.10)	0.23
T2DM	3 (771)	0.15	0.01 (−0.23 to 0.26)	0.91
Healthy subjects	10 (1 717)	0.44	−0.09 (−0.19 to 0.02)	0.10
**HDL-C**				
All	36 (7 287)	<0.001	0.07 (−0.03 to 0.16)	0.17
Studies in HWE	23 (4 795)	<0.001	0.07 (−0.05 to 0.19)	0.25
Caucasian	9 (2 850)	<0.01	−0.01 (−0.18 to 0.16)	0.91
Asian	13 (2 678)	<0.001	0.14 (−0.02 to 0.31)	0.09
Turkish	6 (791)	<0.001	0.10 (−0.23 to 0.43)	0.54
Other ethnicity	8 (968)	0.20	−0.00 (−0.17 to 0.17)	0.97
Female	5 (874)	0.11	0.26 (0.03 to 0.48)	0.03
CAD	4 (554)	<0.01	0.07 (−0.32 to 0.46)	0.72
T2DM	5 (1 984)	0.08	−0.03 (−0.19 to 0.14)	0.74
Healthy subjects	12 (1 873)	<0.001	0.09 (−0.12 to 0.30)	0.40

*SERPINE1, plasminogen activator inhibitor-1; SMD, standardized mean difference; 95% CI, 95% confidence interval; HWE, Hardy-Weinberg equilibrium; CAD, coronary artery disease; T2DM, type 2 diabetes mellitus; TG, triglycerides; TC, total cholesterol; LDL-C, low-density lipoprotein cholesterol; HDL-C, high-density lipoprotein cholesterol.*

**FIGURE 5:**
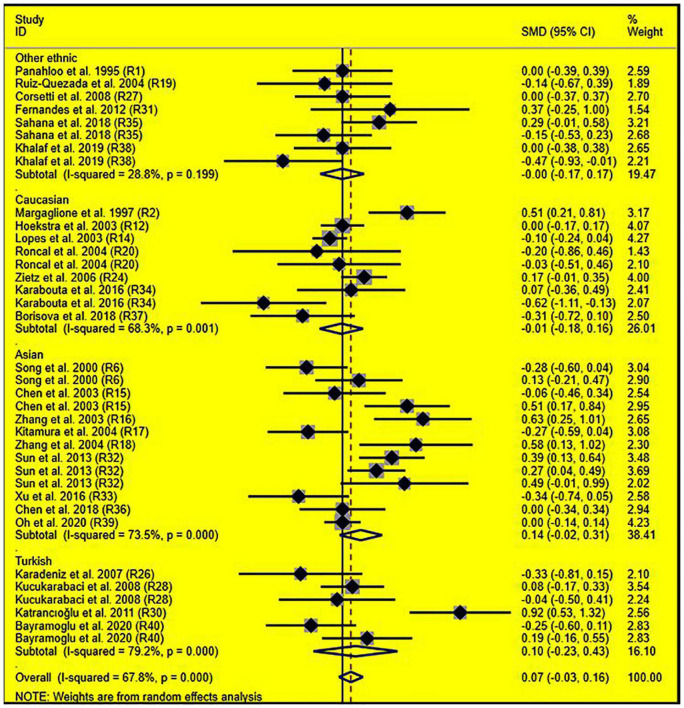
Forest plot of the meta-analysis between *SERPINE1* rs1799889 polymorphism and circulating HDL-C levels.

Subgroup analysis by ethnicity showed that the significant effects of the rs1799889 variant on LDL-C (SMD = –0.15, 95% CI = –0.26 to 0.05, *P* < 0.01) and TC (SMD = –0.17, 95% CI = –0.27 to 0.07, *P* < 0.01) levels were only observed in the Asian population. Meanwhile, the significant effect of the rs1799889 variant on TG (SMD = –0.31, 95% CI = –0.59 to 0.04, *P* = 0.03) level was only observed in the Turkish population. Moreover, a marginally significant effect of the rs1799889 variant on TG (SMD = –0.09, 95% CI = –0.17 to 0.00, *P* = 0.06) and HDL-C [SMD = 0.14, 95% CI = –0.02 to 0.31, *P* = 0.09; Figure 5] levels was observed in the Asian population (Table 1). Subgroup analysis by gender showed that the significant effect of the rs1799889 variant on HDL-C level was only detected in female subjects (Table 1).

The analyses were re-performed after excluding the studies with heterogeneity (Table 2). Notably, the effects of the rs1799889 variant on lipid levels did not change substantially (Table 2), indicating the synthetic results (the analysis data reflect the effect of the rs1799889 variant on lipid profile at total or subgroup level) were robust (refer to Table 2 for more details).

**TABLE 2:** Meta-analysis of *SERPINE1* rs1799889 polymorphism with lipid levels (after eliminating the studies with heterogeneity).

Groups or subgroups	Comparisons (Subjects)	*P*_H_	SMD (95% CI)	*P*_SMD_
**TG**				
All	38 (9 749)	0.43	−0.05 (−0.09 to −0.00)	0.04
Studies in HWE	27 (7 763)	0.38	0.05 (0.02 to 0.09)	<0.01
Caucasian	12 (5 105)	0.23	−0.02 (−0.09 to 0.04)	0.43
Asian	18 (3 647)	0.35	−0.05 (−0.12 to 0.02)	0.18
Turkish	3 (349)	0.80	−0.17 (−0.39 to 0.05)	0.13
Other ethnic	5 (648)	0.73	−0.13 (−0.30 to 0.04)	0.14
Male	2 (461)	0.05	−0.06 (−0.26 to 0.14)	0.58
Female	6 (1 118)	0.47	−0.12 (−0.26 to 0.01)	0.07
CAD	5 (595)	0.80	−0.10 (−0.26 to 0.07)	0.25
T2DM	5 (1 288)	0.13	−0.11 (−0.22 to 0.01)	0.08
Healthy subjects	12 (4 621)	0.55	−0.01 (−0.08 to 0.05)	0.73
**TC**				
All	45 (9 283)	0.37	−0.08 (−0.13 to −0.04)	<0.001
Studies in HWE	30 (6 226)	0.50	−0.07 (−0.13 to −0.02)	0.01
Caucasian	13 (3 753)	0.25	−0.06 (−0.13 to 0.01)	0.09
Asian	18 (3 771)	0.31	−0.09 (−0.16 to −0.02)	0.01
Turkish	6 (791)	0.24	−0.13 (−0.27 to 0.01)	0.08
Other ethnic	8 (968)	0.71	−0.07 (−0.21 to 0.07)	0.33
Male	2 (461)	0.70	−0.06 (−0.26 to 0.14)	0.57
Female	7 (1 175)	0.34	−0.08 (−0.21 to 0.05)	0.23
CAD	4 (509)	0.03	−0.13 (−0.32 to 0.05)	0.15
T2DM	8 (1 698)	0.30	−0.07 (−0.18 to 0.03)	0.17
Healthy subjects	14 (3 270)	0.69	−0.09 (−0.17 to −0.02)	0.02
**LDL-C**				
All	24 (3 662)	0.41	−0.11 (−0.18 to −0.04)	<0.01
Studies in HWE	14 (1 579)	0.38	−0.10 (−0.15 to −0.05)	<0.01
Caucasian	3 (806)	0.62	−0.07 (−0.22 to 0.08)	0.39
Asian	11 (1 737)	0.36	−0.15 (−0.25 to −0.05)	<0.01
Turkish	4 (458)	0.13	−0.09 (−0.29 to 0.10)	0.34
Other ethnicity	6 (661)	0.45	−0.05 (−0.22 to 0.12)	0.55
Female	5 (874)	0.88	−0.14 (−0.31 to 0.03)	0.16
CAD	4 (554)	0.08	0.03 (−0.07 to 0.12)	0.10
T2DM	2 (224)	0.96	−0.15 (−0.42 to 0.13)	0.29
Healthy subjects	10 (1 717)	0.44	−0.09 (−0.19 to 0.02)	0.10
**HDL-C**				
All	28 (5 897)	0.12	−0.02 (−0.08 to 0.03)	0.41
Studies in HWE	19 (4 238)	0.05	−0.03 (−0.12 to 0.07)	0.57
Caucasian	7 (2 554)	0.23	−0.02 (−0.10 to 0.07)	0.70
Asian	8 (1 693)	0.10	−0.05 (−0.15 to 0.05)	0.33
Turkish	5 (682)	0.27	−0.02 (−0.17 to 0.14)	0.81
Other ethnicity	8 (968)	0.20	0.01 (−0.13 to 0.15)	0.89
Female	3 (238)	0.05	0.14 (−0.17 to 0.44)	0.37
CAD	3 (468)	0.02	−0.05 (−0.24 to 0.14)	0.60
T2DM	5 (1 984)	0.08	−0.02 (−0.12 to 0.07)	0.64
Healthy subjects	9 (1 526)	0.34	0.00 (−0.11 to 0.11)	0.99

*SERPINE1, plasminogen activator inhibitor-1; SMD, standardized mean difference; 95% CI, 95% confidence interval; HWE, Hardy-Weinberg equilibrium; CAD, coronary artery disease; T2DM, type 2 diabetes mellitus; TG, triglycerides; TC, total cholesterol; LDL-C, low-density lipoprotein cholesterol; HDL-C, high-density lipoprotein cholesterol.*

### Evaluation of Heterogeneity

In analyzing the effects of the rs1799889 variant on lipid profile, significant heterogeneity was observed (Table 1). Seven (Seguí, 2000; van Harmelen, 2000; Lopes, 2003; Wang, 2007; Katrancıoğlu, 2011; Borisova, 2018; Khalaf, 2019), two (Zhang, 2003, 2004), two (Zietz, 2006; Borisova, 2018), and eight (Margaglione, 1997; Chen, 2003; Zhang, 2003, 2004; Katrancıoğlu, 2011; Sun, SK1 2013, Sun SK2 2013, Karabouta, 2016) comparisons (refer to Supplementary Material for more details about reference citations) were identified as the potential sources of heterogeneity to TG, TC, LDL-C, and HDL-C, respectively. However, the recalculated results did not change significantly after excluding these comparisons (refer to Table 2 for more details).

### Publication Bias Test

There was no publication bias in the synthetic results (refer to Supplementary Figures 1–4 for more details), indicating the synthetic results were reliable.

## Discussion

Our data showed that the rs1799889 variant had significant effects on circulating TG, TC, and LDL-C levels. Subgroup analysis further indicated the significant effects of the rs1799889 variant on TC and LDL-C levels primarily in the Asian population.

Several underlying mechanisms could be proposed to explain the effects of the rs1799889 variant on lipids levels. (1) By repressing peroxisome proliferator-activated receptor γ (PPARγ) expression: PPARγ, a known adipogenic gene (20), plays a major role in TG synthesis (21), the inhibition of PAI-1 activity caused by rs1799889 (1, 2) may repress the expression of PPARγ (3). As a result, the synthesis of TG was reduced; accordingly, the plasma levels of TG were decreased (Tables 1, 2). (2) By disturbing the expression of PCSK9: PCSK9 plays a central role in the degradation of LDLR; the decreased PAI-1 activity caused by rs1799889 (1, 2) may reduce PCSK9 expression (6), therefore, upregulating LDLR; therefore, circulating LDL-C levels was decreased (Tables 1, 2). (3) By modulating the expression of low-density lipoprotein receptor-related protein (LRP). SERPINE1 contains a high-affinity binding site for LRP (22, 23), and the fulfilling of some important physiological functions depends on the cooperation between SERPINE1 and LRP (24, 25). Therefore, the largely decreased PAI-1 activity caused by rs1799889 (1, 2) may affect the expression of LRP.

This analysis indicated the 5G allele of the rs1799889 variant significantly decreased TG, TC, and LDL-C levels (Tables 1, 2), when combined with previous meta-analyses (7–10), indicating the 5G allele of the rs1799889 variant was a causal genetic marker for decreased CAD risk.

According to the 2018 ACC/AHA (26), the 2019 ESC/EAS (27), and the adult treatment panel III (ATP III) cholesterol guidelines (28), LDL-C was regarded as the major initiating factor for CAD and used as the main intervention target, while TC, HDL-C, and TG were used as the secondary targets. In this study, significantly reduced LDL-C levels were observed in subjects with the rs1799889 variant (Tables 1, 2), indicating the 4G/5G insertion/deletion sequence of *SERPINE1* potentially be the target for CAD therapy. Moreover, subgroup analysis by ethnicity showed that the significant effects of the rs1799889 variant on LDL-C and TC levels were primarily in Asians (Tables 1, 2), indicating the Asians with the 5G allele of the rs1799889 variant may have a reduced CAD risk. Intriguingly, this speculation was verified by Li et al. (29).

### Strengths and Limitations

To the best of our knowledge, this is the first meta-analysis to investigate the effects of the *SERPINE1* 4G/5G insertion/deletion sequence on circulating lipid levels. Several strengths of the meta-analysis should be noted. For instance, all data were recalculated after excluding the studies with heterogeneity, which no doubt advances the preciseness of conclusions drawn in this manuscript. In addition, the analysis results benefit insight into the mechanism underlying the rs1799889 variant and CAD. More importantly, our data indicate that the 4G/5G insertion/deletion sequence of the *SERPINE1* gene potentially be the target for CAD therapy. However, this meta-analysis did not investigate the interaction between the rs1799889 variant and environmental factors on lipid levels due to the lack of available data from the included studies.

## Conclusion

The rs1799889 variant of *SERPINE1* is a protective genetic factor against CAD, and the Asian population with the 5G allele of the rs1799889 variant may have a reduced CAD risk.

## Data Availability Statement

The original contributions presented in this study are included in the article/Supplementary Material, further inquiries can be directed to the corresponding authors.

## Author Contributions

ZL, BW, JW, and YL conceived and designed this study as well as drafted the manuscript and performed the statistical analyses. HL, YZ, YP, XL, and YF carried out the searches and collected the data. All authors reviewed and approved the final manuscript.

## Conflict of Interest

The authors declare that the research was conducted in the absence of any commercial or financial relationships that could be construed as a potential conflict of interest.

## Publisher’s Note

All claims expressed in this article are solely those of the authors and do not necessarily represent those of their affiliated organizations, or those of the publisher, the editors and the reviewers. Any product that may be evaluated in this article, or claim that may be made by its manufacturer, is not guaranteed or endorsed by the publisher.
