# Supplementary material for: Systematic Review and Meta-Analysis of SERPINE1 4G/5G Insertion/Deletion Variant With Circulating Lipid Levels
Source: Front Cardiovasc Med. 2022 Jun 23;9:859979. doi: 10.3389/fcvm.2022.859979 (PMC9260103; doi:10.3389/fcvm.2022.859979)
Supplement: Supplementary file 2 [file Data_Sheet_1.docx]

Supplementary Material

**Supplemental Tables:**

**Table S1.** Characteristics of the included studies.

**Table S2.** Circulating lipid levels by the genotypes of *SERPINE1* rs1799889 polymorphism.

**Supplemental Figures:**

**Figure S1.** Begg’s funnel plot of the association analysis between *SERPINE1* rs1799889 polymorphism and circulating TG levels.

**Figure S2.** Begg’s funnel plot of the association analysis between *SERPINE1* rs1799889 polymorphism and circulating TC levels.

**Figure S3.** Begg’s funnel plot of the association analysis between *SERPINE1* rs1799889 polymorphism and circulating LDL-C levels.

**Figure S4.** Begg’s funnel plot of the association analysis between *SERPINE1* rs1799889 polymorphism and circulating HDL-C levels.

**Table S1.** Characteristics of the included studies.

| **First author, reference** | **Year** | **Ethnicity** | **Gender** | **Study population** | **Outcomes** |
| --- | --- | --- | --- | --- | --- |
| Panahloo A [R1] | 1995 | Other ethnic | M/F | Patients with T2DM | TC/HDL |
| Margaglione M [R2] | 1997 | Caucasian | M/F | Patients with high risk of CAD | HDL |
| Margaglione M [R3] | 1998 | Caucasian | M/F | Healthy subjects | TG/TC/LDL/HDL |
| Burzotta F [R4] | 1998 | Caucasian | M/F | Healthy subjects | TG/TC |
| Grancha S [R5] | 1999 | Caucasian | F | Patients with CAD | TG/TC |
| Song JH1 [R6] | 2000 | Asian | M/F | Patients with CAD | TG/TC/LDL/HDL |
| Song JH2 [R6] | 2000 | Asian | M/F | Control subjects | TG/TC/LDL/HDL |
| Wong TY [R7] | 2000 | Asian | M/F | Patients with T2DM | TG/TC |
| Seguí R [R8] | 2000 | Caucasian | M/F | Patients with deep vein thrombosis | TG/TC |
| van Harmelen V [R9] | 2000 | Caucasian | M/F | Healthy subjects | TG |
| Estellés A [R10] | 2001 | Caucasian | M/F | Patients with obesity | TG |
| Li CG [R11] | 2001 | Asian | M/F | Patients with T2DM | TG/TC |
| Hoekstra T [R12] | 2003 | Caucasian | M/F | Healthy subjects | TC/LDL/HDL |
| Jeng JR1 [R13] | 2003 | Asian | M | Patients with hypertension and healthy subjects | TG/TC |
| Jeng JR2 [R13] | 2003 | Asian | F | Patients with hypertension and healthy subjects | TG/TC |
| Lopes C [R14] | 2003 | Caucasian | M/F | Patients with T2DM | TG/HDL |
| Chen CH1 [R15] | 2003 | Asian | M/F | Patients with stroke | TG/TC/LDL/HDL |
| Chen CH2 [R15] | 2003 | Asian | M/F | Control subjects | TG/TC/LDL/HDL |
| Zhang AJ [R16] | 2003 | Asian | M/F | Patients with cerebral infarction | TG/TC/LDL/HDL |
| Kitamura Y [R17] | 2004 | Asian | M | Healthy subjects | TG/TC/LDL/HDL |
| Zhang AY [R18] | 2004 | Asian | M/F | Patients with CAD | TG/TC/LDL/HDL |
| Ruiz-Quezada S [R19] | 2004 | Other ethnic | M/F | Healthy subjects | TG/TC/LDL/HDL |
| Roncal C1 [R20] | 2004 | Caucasian | M/F | Patients with hypertension | TG/TC/HDL |
| Roncal C2 [R20] | 2004 | Caucasian | M/F | Control subjects | TG/TC/HDL |
| Liu SQ [R21] | 2004 | Asian | M/F | Patients with T2DM | TG/TC/LDL/HDL |
| Chen YL [R22] | 2005 | Asian | M/F | Patients with deep vein thrombosis | TG/TC/LDL/HDL |
| Meigs JB [R23] | 2006 | Caucasian | M/F | Healthy subjects | TG |
| Zietz B [R24] | 2006 | Caucasian | M/F | Patients with T2DM | TG/TC/LDL/HDL |
| Wang L [R25] | 2007 | Asian | M/F | Patients with T2DM and control subjects | TG/TC |
| Karadeniz M [R26] | 2007 | Turkish | F | Patients with PCOS | TG/TC/LDL/HDL |
| Corsetti JP [R27] | 2008 | Other ethnic | M/F | Patients with high risk of CAD | TG/TC/LDL/HDL |
| Kucukarabaci B1 [R28] | 2008 | Turkish | M/F | Patients with stroke | TC/HDL |
| Kucukarabaci B2 [R28] | 2008 | Turkish | M/F | Control subjects | TC/HDL |
| Wang HJ1 [R29] | 2011 | Caucasian | M/F | Patients with T1DM | TG/TC |
| Wang HJ2 [R29] | 2011 | Caucasian | M/F | Patients with T2DM | TG/TC |
| Katrancıoğlu N [R30] | 2011 | Turkish | M/F | Healthy subjects | TG/TC/LDL/HDL |
| Fernandes KS [R31] | 2012 | Other ethnic | F | Patients with obesity | TC/LDL/HDL |
| Sun SK1 [R32] | 2013 | Asian | F | Patients with metabolic syndrome | TG/TC/LDL/HDL |
| Sun SK2 [R32] | 2013 | Asian | F | Patients with non-metabolic syndrome | TG/TC/LDL/HDL |
| Sun SK3 [R32] | 2013 | Asian | F | Control subjects | TG/TC/LDL/HDL |
| Xu F [R33] | 2016 | Asian | M/F | Patients with T2DM | TG/TC/LDL/HDL |
| Karabouta Z1 [R34] | 2016 | Caucasian | M/F | Patients with obesity | TG/TC/LDL/HDL |
| Karabouta Z2 [R34] | 2016 | Caucasian | M/F | Control subjects | TG/TC/LDL/HDL |
| Sahana N1 [R35] | 2018 | Other ethnic | M/F | Patients with CAD | TG/TC/LDL/HDL |
| Sahana N2 [R35] | 2018 | Other ethnic | M/F | Control subjects | TG/TC/LDL/HDL |
| Chen TH [R36] | 2018 | Asian | M/F | Patients with adenoma | TG/TC/HDL |
| Borisova EP [R37] | 2018 | Caucasian | M/F | Patients with COPD | TG/TC/LDL/HDL |
| Khalaf FA1 [R38] | 2019 | Other ethnic | M/F | Patients with T2DM | TG/TC/LDL/HDL |
| Khalaf FA2 [R38] | 2019 | Other ethnic | M/F | Patients with T2DM and vascular complication | TG/TC/LDL/HDL |
| Oh J [R39] | 2020 | Asian | M/F | Patients with colorectal cancer | TG/TC/HDL |
| Bayramoglu A1 [R40] | 2020 | Turkish | M/F | Patients with CAD | TG/TC/LDL/HDL |
| Bayramoglu A2 [R40] | 2020 | Turkish | M/F | Control subjects | TG/TC/LDL/HDL |

M: male; F: female; CAD: coronary artery disease; T1DM: type 1 diabetes mellitus; T2DM: type 2 diabetes mellitus; COPD: chronic obstructive pulmonary disease; PCOS: polycystic ovary syndrome; TG: triglycerides; TC: total cholesterol; LDL-C: low-density lipoprotein cholesterol; HDL-C: high-density lipoprotein cholesterol.

**Table S2.** Circulating lipid levels by the genotypes of *SERPINE1* rs1799889 polymorphism.

| **First author, reference** | **Number** | |  | **TG, mmol/L** | |  | **TC, mmol/L** | |  | **LDL-C, mmol/L** | |  | **HDL-C, mmol/L** | |
| --- | --- | --- | --- | --- | --- | --- | --- | --- | --- | --- | --- | --- | --- | --- |
|  | **4G/4G** | **4G/5G+5G/5G** |  | **4G/4G** | **4G/5G+5G/5G** |  | **4G/4G** | **4G/5G+5G/5G** |  | **4G/4G** | **4G/5G+5G/5G** |  | **4G/4G** | **4G/5G+5G/5G** |
| Panahloo A [R1] | 32 | 114 |  | 1.33±0.91 | 1.28±0.88 |  | 5.6±0.8 | 5.25±1.11 |  | - | - |  | 1.1±0.3 | 1.1±0.3 |
| Margaglione M [R2] | 62 | 146 |  | 1.2±0.57 | 1.33±0.66 |  | - | - |  | - | - |  | 0.76±0.21 | 0.87±0.22 |
| Margaglione M [R3] | 307 | 872 |  | 1.71±0.81 | 1.42±0.64 |  | 4.95±0.94 | 4.85±1.01 |  | - | - |  | - | - |
| Burzotta F [R4] | 72 | 146 |  | 1.5±0.75 | 1.54±0.86 |  | 5.15±1.03 | 5.11±1.07 |  | - | - |  | - | - |
| Grancha S [R5] | 6 | 35 |  | 1.26±0.48 | 1.26±0.57 |  | 6.31±1.47 | 5.5±0.98 |  | - | - |  | - | - |
| Song JH1 [R6] | 62 | 96 |  | 2.34±1.44 | 1.87±1.96 |  | 5.42±1.03 | 4.99±1.01 |  | 3.54±0.97 | 3.19±0.89 |  | 1.19±0.36 | 1.1±0.29 |
| Song JH2 [R6] | 54 | 85 |  | 1.2±0.5 | 1.56±1 |  | 5.24±0.93 | 5.36±0.84 |  | 3.15±0.82 | 3.21±0.79 |  | 1.52±0.33 | 1.57±0.42 |
| Wong TY [R7] | 47 | 94 |  | 1.8±0.2 | 1.54±0.14 |  | 5.8±1.0 | 5.54±1.2 |  | - | - |  | - | - |
| Seguí R [R8] | 40 | 150 |  | 0.86±0.59 | 0.88±0.42 |  | 5.4±1.1 | 5.08±1.07 |  | - | - |  | - | - |
| van Harmelen V [R9] | 24 | 65 |  | 1.79±0.7 | 1.86±0.78 |  | - | - |  | - | - |  | - | - |
| Estellés A [R10] | 29 | 73 |  | - | - |  | - | - |  | - | - |  | - | - |
| Li CG [R11] | 52 | 91 |  | 1.93±2.43 | 1.6±1.03 |  | 5.38±1.05 | 5.24±1.07 |  | - | - |  | - | - |
| Hoekstra T [R12] | 193 | 436 |  | 1.34±0.85 | 1.41±1.15 |  | 6.3±1.2 | 6.2±1.23 |  | 3.8±1.1 | 3.73±1.0 |  | 1.4±0.5 | 1.4±0.37 |
| Jeng JR1 [R13] | 77 | 228 |  | 1.29±0.9 | 1.39±0.9 |  | 5.25±1.17 | 5.16±0.93 |  | - | - |  | - | - |
| Jeng JR2 [R13] | 69 | 191 |  | 1.98±1.5 | 1.94±1.1 |  | 5.38±0.82 | 5.42±1.1 |  | - | - |  | - | - |
| Lopes C [R14] | 288 | 779 |  | 1.69±0.99 | 1.45±0.83 |  | - | - |  | - | - |  | 1.49±0.4 | 1.45±0.4 |
| Chen CH1 [R15] | 40 | 60 |  | 2.1±0.86 | 1.98±1.05 |  | 5.43±0.95 | 5.37±1.07 |  | 3.37±0.71 | 3.35±0.84 |  | 1.15±0.29 | 1.13±0.34 |
| Chen CH2 [R15] | 58 | 92 |  | 1.56±1.09 | 1.84±1.85 |  | 5.49±1.23 | 5.28±1.07 |  | 3.4±1.11 | 3.11±1 |  | 1.32±0.31 | 1.51±0.41 |
| Zhang AJ [R16] | 48 | 65 |  | 2.08±0.90 | 1.95±1.07 |  | 5.73±1.02 | 5.05±0.6 |  | 3.15±0.83 | 2.82±0.68 |  | 1.15±0.26 | 1.3±0.22 |
| Kitamura Y [R17] | 77 | 79 |  | 1.46±0.89 | 1.52±0.97 |  | 5.15±0.90 | 5.14±0.99 |  | 3.09±0.84 | 3.15±0.97 |  | 1.42±0.37 | 1.33±0.29 |
| Zhang AY [R18] | 33 | 53 |  | 1.21±0.35 | 1.62±0.87 |  | 5.63±1.09 | 4.98±0.68 |  | 3.14±0.88 | 2.8±0.7 |  | 1.16±0.29 | 1.31±0.24 |
| Ruiz-Quezada S [R19] | 16 | 94 |  | 1.23±0.6 | 1.3±0.79 |  | 5.17±1.34 | 5.1±1.03 |  | 3.41±1.03 | 3.28±0.91 |  | 1.22±0.44 | 1.17±0.34 |
| Roncal C1 [R20] | 12 | 34 |  | - | - |  | 6.21±0.69 | 6.59±1.1 |  | - | - |  | 1.35±0.34 | 1.29±0.28 |
| Roncal C2 [R20] | 24 | 52 |  | 1.48±0.53 | 1.57±0.35 |  | 6.09±1.1 | 6.13±1 |  | - | - |  | 1.42±0.35 | 1.41±0.38 |
| Liu SQ [R21] | 42 | 105 |  | 1.05±0.85 | 1.06±0.86 |  | 5.8±1.33 | 5.29±1.25 |  | - | - |  | - | - |
| Chen YL [R22] | 46 | 74 |  | 3.69±2.27 | 3.29±1.91 |  | 4.92±1.07 | 4.8±0.49 |  | - | - |  | - | - |
| Meigs JB [R23] | 594 | 1575 |  | 2.24±0.68 | 1.95±0.46 |  | - | - |  | - | - |  | - | - |
| Zietz B [R24] | 176 | 371 |  | 1.57±0.91 | 1.34±0.74 |  | 6.85±1.32 | 6.9±1.32 |  | 4.01±1.19 | 4.22±1.21 |  | 1.14±0.34 | 1.2±0.36 |
| Wang L [R25] | 68 | 108 |  | 1.58±0.25 | 1.5±0.32 |  | 5.6±2.31 | 4.87±2.21 |  | - | - |  | - | - |
| Karadeniz M [R26] | 22 | 67 |  | - | - |  | 5.24±0.89 | 5.09±1.15 |  | 3±0.58 | 3.03±0.91 |  | 1.58±0.55 | 1.45±0.33 |
| Corsetti JP [R27] | 35 | 126 |  | - | - |  | 4.13±0.59 | 4.04±0.51 |  | - | - |  | 0.94±0.22 | 0.94±0.21 |
| Kucukarabaci B1 [R28] | 114 | 139 |  | 1.1±1.12 | 0.93±0.69 |  | 4.66±1.26 | 4.76±1.4 |  | - | - |  | 1.2±0.43 | 1.23±0.35 |
| Kucukarabaci B2 [R28] | 29 | 51 |  | 1.8±1.31 | 1.95±1.83 |  | 5.33±1.17 | 4.9±1.11 |  | - | - |  | 1.34±0.25 | 1.3±1.15 |
| Wang HJ1 [R29] | 62 | 138 |  | 2.1±0.57 | 1.75±0.42 |  | 5.02±1.18 | 5.07±1.31 |  | - | - |  | - | - |
| Wang HJ2 [R29] | 112 | 238 |  | - | - |  | 4.9±1.4 | 4.96±1.33 |  | - | - |  | - | - |
| Katrancıoğlu N [R30] | 50 | 59 |  | 2.56±1.68 | 2.17±1.31 |  | 4.77±1.17 | 4.27±0.98 |  | 3.42±0.82 | 3.04±0.73 |  | 0.8±0.08 | 0.89±0.11 |
| Fernandes KS [R31] | 13 | 44 |  | 1.34±0.68 | 1.27±0.58 |  | 4.62±0.94 | 4.4±1.09 |  | 2.82±1.01 | 2.57±0.67 |  | 1.12±0.31 | 1.23±0.29 |
| Sun SK1 [R32] | 88 | 184 |  | 0.89±0.31 | 0.91±0.31 |  | 5.5±1.2 | 5.6±0.92 |  | 3±0.8 | 2.88±0.73 |  | 1.32±0.24 | 1.47±0.44 |
| Sun SK2 [R32] | 107 | 257 |  | 4.69±1.33 | 4.09±2.05 |  | 5.6±1 | 5.38±0.95 |  | 2.9±0.8 | 2.84±0.64 |  | 1.59±0.31 | 1.69±0.4 |
| Sun SK3 [R32] | 20 | 72 |  | 1.12±0.61 | 1.04±0.58 |  | 5.5±0.8 | 5.45±0.83 |  | 2.9±0.3 | 2.9±0.52 |  | 1.63±0.28 | 1.77±0.29 |
| Xu F [R33] | 40 | 67 |  | 0.85±0.47 | 0.76±0.32 |  | 4.69±1.33 | 4.54±1.41 |  | 3.06±0.85 | 2.93±0.95 |  | 1.13±0.49 | 1±0.29 |
| Karabouta Z1 [R34] | 36 | 53 |  | 4.58±2.48 | 4.31±2.61 |  | 4.27±1.01 | 4.11±0.72 |  | 2.57±0.88 | 2.42±0.66 |  | 1.19±0.29 | 1.21±0.3 |
| Karabouta Z2 [R34] | 22 | 66 |  | 4.83±2.25 | 4.34±1.31 |  | 4.3±0.67 | 4.52±0.93 |  | 2.45±0.57 | 2.55±0.87 |  | 1.46±0.34 | 1.11±0.62 |
| Sahana N1 [R35] | 101 | 79 |  | 1.45±0.62 | 1.38±0.73 |  | 5.12±1.59 | 5.36±1.58 |  | 3.43±1.80 | 3.5±1.71 |  | 1.09±0.33 | 1.18±0.29 |
| Sahana N2 [R35] | 70 | 44 |  | 1.6±0.11 | 1.38±0.09 |  | 5.69±3.44 | 5.27±2.68 |  | 3.74±1.15 | 3.49±1.34 |  | 1.02±0.24 | 0.98±0.31 |
| Chen TH [R36] | 53 | 95 |  | 1.52±0.42 | 1.74±0.38 |  | 5.2±1.08 | 5.1±0.88 |  | - | - |  | 1.2±0.25 | 1.2±0.32 |
| Borisova EP [R37] | 37 | 63 |  | 1.87±0.33 | 1.89±0.38 |  | 5.3±0.14 | 5.21±0.21 |  | 3.5±0.12 | 3.33±0.16 |  | 1.4±0.07 | 1.38±0.06 |
| Khalaf FA1 [R38] | 40 | 77 |  | 1.49±0.96 | 1.55±0.92 |  | 5.03±0.65 | 5.03±0.57 |  | 3.33±0.46 | 3.26±0.45 |  | 0.85±0.15 | 0.85±0.22 |
| Khalaf FA2 [R38] | 28 | 55 |  | 2.01±1.32 | 1.8±0.75 |  | 5.11±0.91 | 5.08±0.57 |  | 3.15±0.55 | 3.31±0.47 |  | 1.02±0.1 | 0.96±0.14 |
| Oh J [R39] | 314 | 479 |  | 2.01±1.1 | 1.89±1.47 |  | 4.8±1.06 | 4.83±0.97 |  | - | - |  | 1.13±0.37 | 1.13±0.33 |
| Bayramoglu A1 [R40] | 79 | 51 |  | 1.33±0.91 | 1.28±0.88 |  | 4.83±1.32 | 4.7±1.34 |  | 2.93±1 | 3.01±1.26 |  | 1.02±0.25 | 0.96±0.23 |
| Bayramoglu A2 [R40] | 51 | 79 |  | 1.2±0.57 | 1.33±0.66 |  | 5.05±0.93 | 4.91±1.06 |  | 2.91±0.9 | 2.92±1.02 |  | 1.13±0.37 | 1.21±0.44 |

*SERPINE1*: plasminogen activator inhibitor-1; TG: triglycerides; TC: total cholesterol; LDL-C: low-density lipoprotein cholesterol; HDL-C: high-density lipoprotein cholesterol.


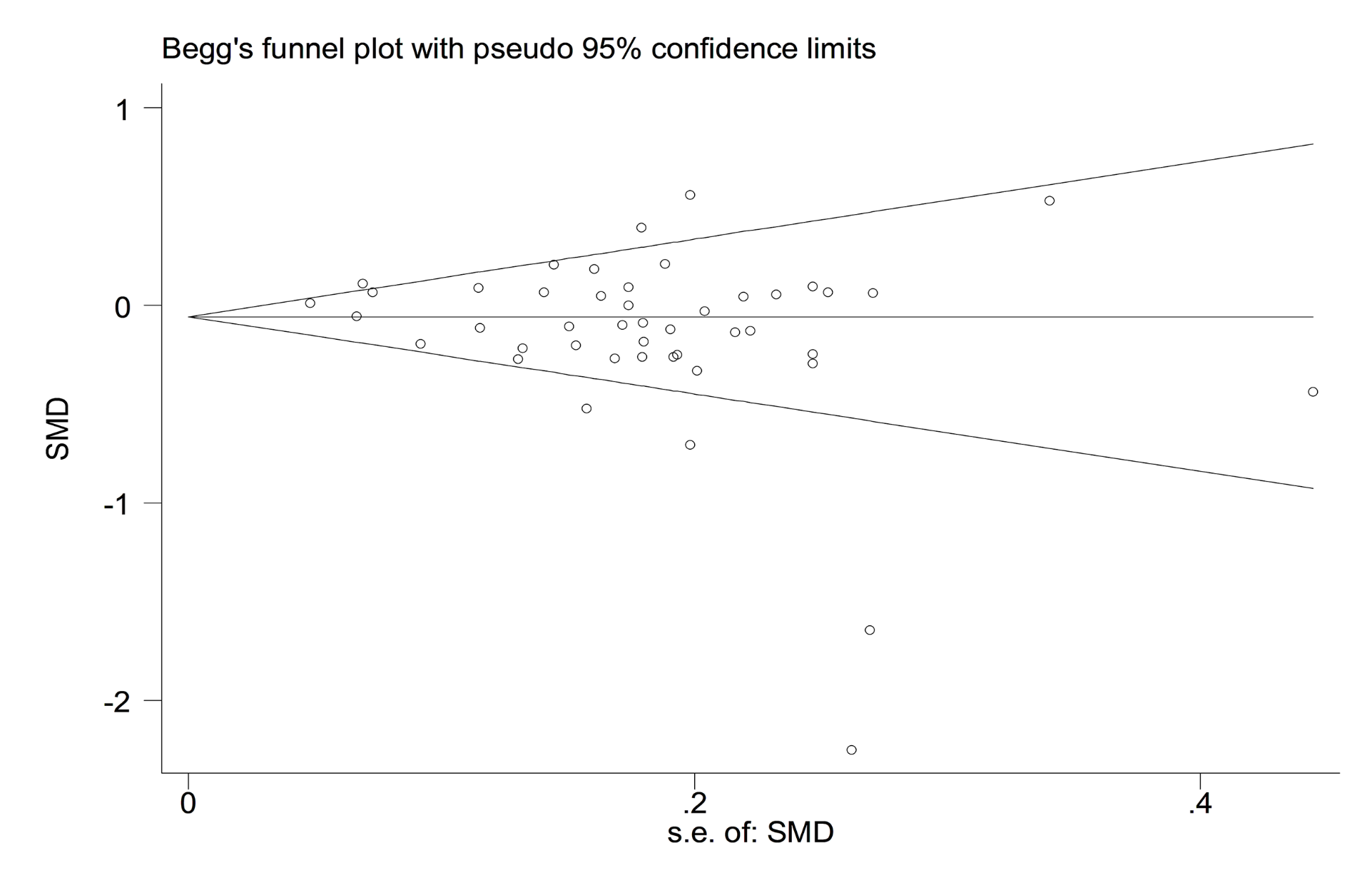


**Figure S1.** Begg’s funnel plot of the association analysis between *SERPINE1* rs1799889 polymorphism and circulating TG levels (*P* = 0.26).


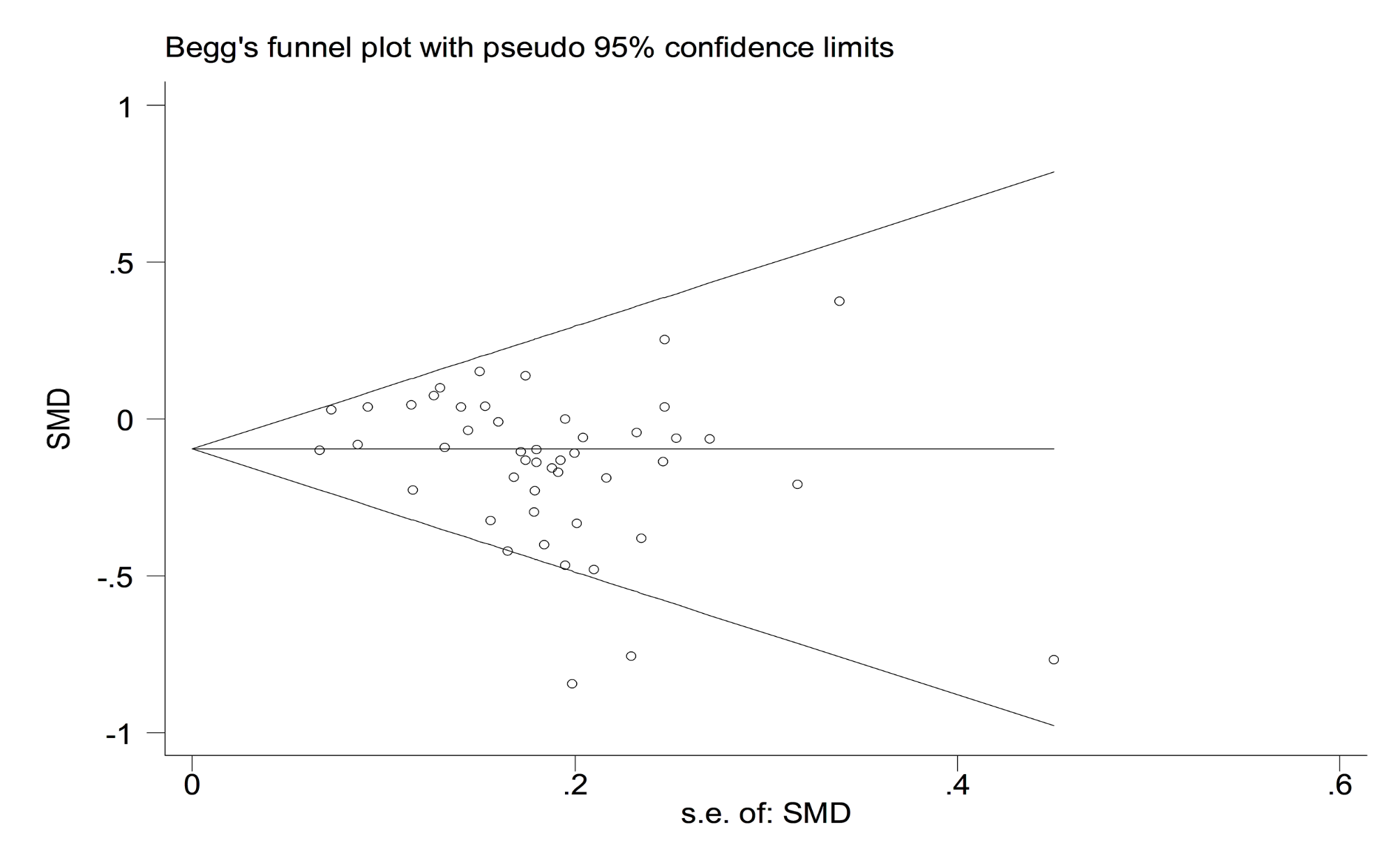


**Figure S2.** Begg’s funnel plot of the association analysis between *SERPINE1* rs1799889 polymorphism and circulating TC levels (*P* = 0.41).


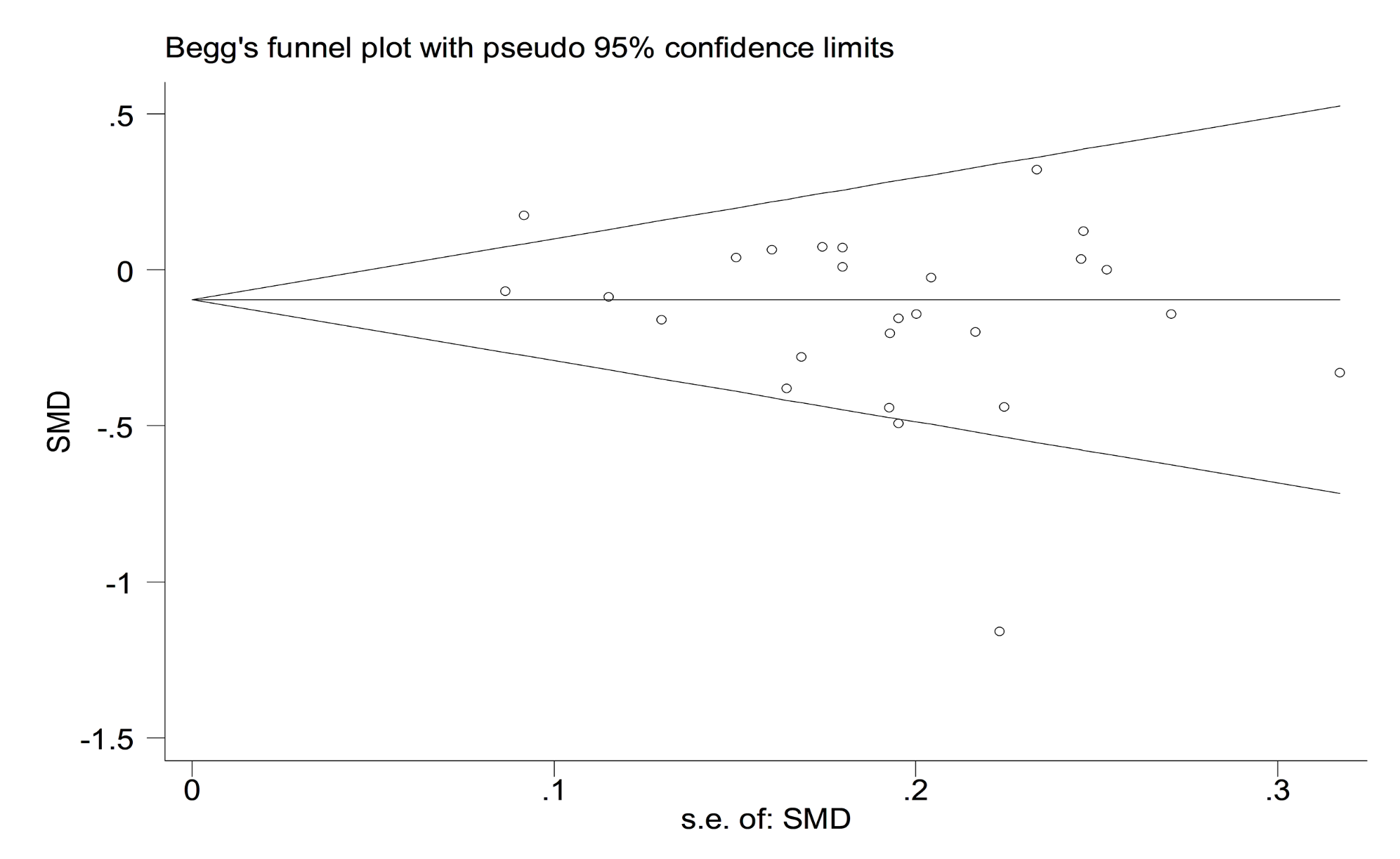


**Figure S3.** Begg’s funnel plot of the association analysis between *SERPINE1* rs1799889 polymorphism and circulating LDL-C levels (*P* = 0.34).


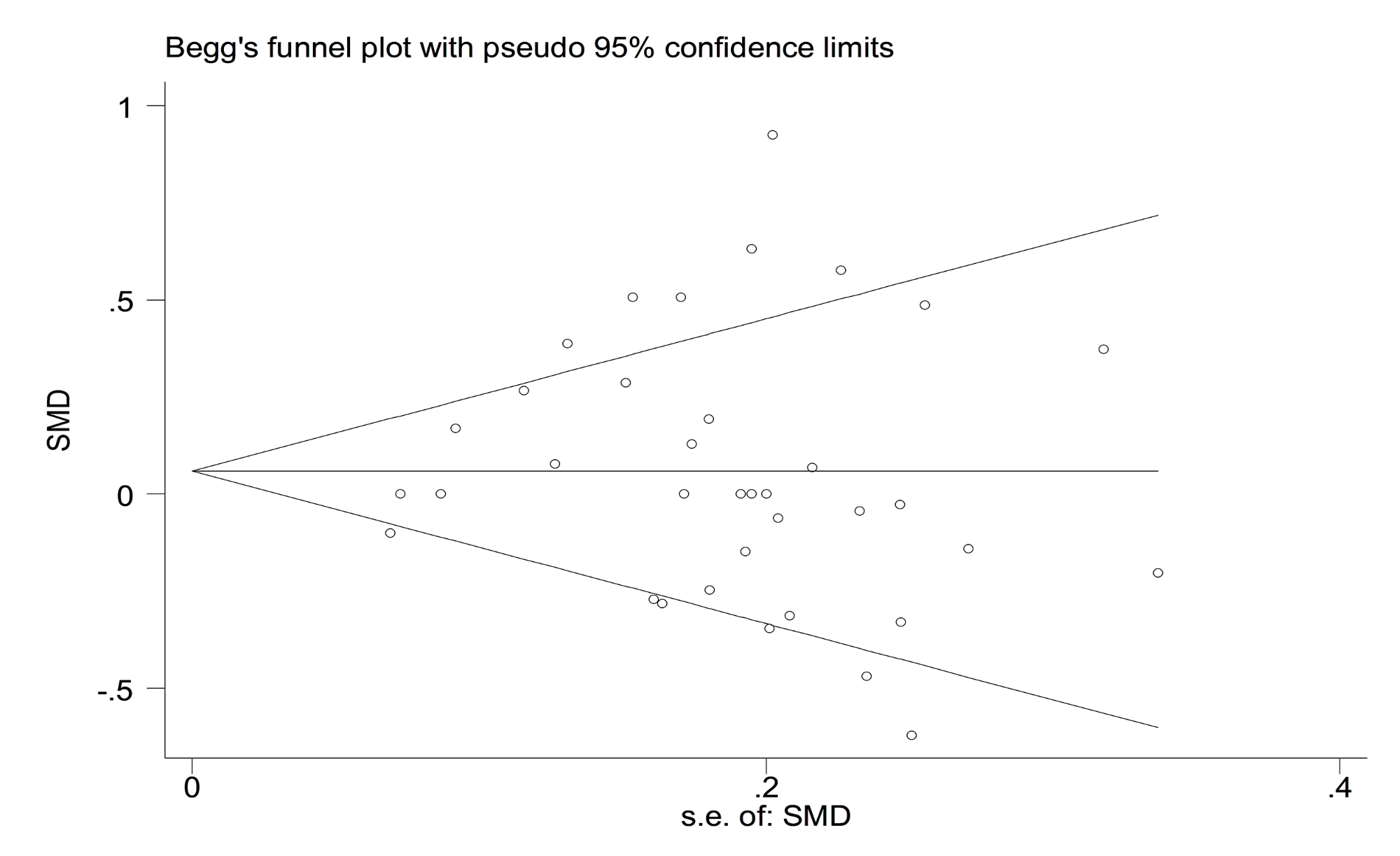


**Figure S4.** Begg’s funnel plot of the association analysis between *SERPINE1* rs1799889 polymorphism and circulating HDL-C levels (*P* = 0.73).

**References of included studies**

R1. Panahloo A, Mohamed-Ali V, Lane A, Green F, Humphries SE, Yudkin JS. Determinants of plasminogen activator inhibitor 1 activity in treated NIDDM and its relation to a polymorphism in the plasminogen activator inhibitor 1 gene. Diabetes. 1995;44(1):37-42. doi: 10.2337/diab.44.1.37.

R2. Margaglione M, Grandone E, Vecchione G, Cappucci G, Giuliani N, Colaizzo D, Celentano E, Panico S, Di Minno G. Plasminogen activator inhibitor-1 (PAI-1) antigen plasma levels in subjects attending a metabolic ward: relation to polymorphisms of PAI-1 and angiontensin converting enzyme (ACE) genes. Arterioscler Thromb Vasc Biol. 1997;17(10):2082-7. doi: 10.1161/01.atv.17.10.2082.

R3. Margaglione M, Cappucci G, Colaizzo D, Giuliani N, Vecchione G, Grandone E, Pennelli O, Di Minno G. The PAI-1 gene locus 4G/5G polymorphism is associated with a family history of coronary artery disease. Arterioscler Thromb Vasc Biol. 1998;18(2):152-6. doi: 10.1161/01.atv.18.2.152.

R4. Burzotta F, Di Castelnuovo A, Amore C, D'Orazio A, Di Bitondo R, Donati MB, Iacoviello L. 4G/5G promoter PAI-1 gene polymorphism is associated with plasmatic PAI-1 activity in Italians: a model of gene-environment interaction. Thromb Haemost. 1998;79(2):354-8.

R5. Grancha S, Estellés A, Tormo G, Falco C, Gilabert J, España F, Cano A, Segui R, Aznar J. Plasminogen activator inhibitor-1 (PAI-1) promoter 4G/5G genotype and increased PAI-1 circulating levels in postmenopausal women with coronary artery disease. Thromb Haemost. 1999;81(4):516-21.

R6. Song J, Yoon YM, Jung HJ, Hong SH, Park H, Kim JQ. Plasminogen activator inhibitor-1 4G/5G promoter polymorphism and coagulation factor VII Arg353-->Gln polymorphism in Korean patients with coronary artery disease. J Korean Med Sci. 2000;15(2):146-52. doi: 10.3346/jkms.2000.15.2.146.

R7. Wong TY, Poon P, Szeto CC, Chan JC, Li PK. Association of plasminogen activator inhibitor-1 4G/4G genotype and type 2 diabetic nephropathy in Chinese patients. Kidney Int. 2000;57(2):632-8. doi: 10.1046/j.1523-1755.2000.00884.x.

R8. Seguí R, Estellés A, Mira Y, España F, Villa P, Falcó C, Vayá A, Grancha S, Ferrando F, Aznar J. PAI-1 promoter 4G/5G genotype as an additional risk factor for venous thrombosis in subjects with genetic thrombophilic defects. Br J Haematol. 2000;111(1):122-8. doi: 10.1046/j.1365-2141.2000.02321.x.

R9. van Harmelen V, Wahrenberg H, Eriksson P, Arner P. Role of gender and genetic variance in plasminogen activator inhibitor-1 secretion from human adipose tissue. Thromb Haemost. 2000;83(2):304-8.

R10. Estellés A, Dalmau J, Falcó C, Berbel O, Castelló R, España F, Aznar J. Plasma PAI-1 levels in obese children--effect of weight loss and influence of PAI-1 promoter 4G/5G genotype. Thromb Haemost. 2001;86(2):647-52.

R11. Li CG, Dong YH, Wang HY, Si YG, Lv WS, Zhai MX, Wang J, Liu L. Association of plasminogen activator inhibitor 4G/5G polymorphism and type 2 diabetes with nephropathy. Chinese Journal of Diabetes. 2001; 9(6): 333-340.

R12. Hoekstra T, Geleijnse JM, Kluft C, Giltay EJ, Kok FJ, Schouten EG. 4G/4G genotype of PAI-1 gene is associated with reduced risk of stroke in elderly. Stroke. 2003;34(12):2822-8. doi: 10.1161/01.STR.0000098004.26252.EB.

R13. Jeng JR. Association of PAI-1 gene promoter 4g/5g polymorphism with plasma PAI-1 activity in Chinese patients with and without hypertension. Am J Hypertens. 2003;16(4):290-6. doi: 10.1016/s0895-7061(03)00004-9.

R14. Lopes C, Dina C, Durand E, Froguel P. PAI-1 polymorphisms modulate phenotypes associated with the metabolic syndrome in obese and diabetic Caucasian population. Diabetologia. 2003;46(9):1284-90. doi: 10.1007/s00125-003-1170-0.

R15. Chen CH, Eng HL, Chang CJ, Tsai TT, Lai ML, Chen HY, Liu CJ, Lin TM. 4G/5G promoter polymorphism of plasminogen activator inhibitor-1, lipid profiles, and ischemic stroke. J Lab Clin Med. 2003;142(2):100-5. doi: 10.1016/S0022-2143(03)00063-5.

R16. Zhang AJ, Song BH, Wang JX, Feng YC. The association between PAI-1 4G/5G polymorphism and cerebral infarction. Shandong Medical Journal. 2003; 43(31): 42-43.

R17. Kitamura Y, Okumura K, Imamura A, Mizuno T, Tsuzuki M, Numaguchi Y, Matsui H, Murohara T. Association of plasminogen activator inhibitor-1 4G/5G gene polymorphism with variations in the LDL particle size in healthy Japanese men. Clin Chim Acta. 2004;347(1-2):209-16. doi: 10.1016/j.cccn.2004.04.014.

R18. Zhang AY, Ji XW, Guan LX, Wang JX, Zhao CH, Zhao LM, Zhang YZ. The relationship between 4G/5G polymorphism of PAI-1 gene and blood lipid and blood sugar in patients with coronary heart disease. 2004; 2(7): 538-539.

R19. Ruiz-Quezada S, Vázquez-Del Mercado M, Parra-Rojas I, Rangel-Villalobos H, Best-Aguilera C, Sánchez-Orozco LV, Muñoz-Valle JF. Genotype and allele frequency of PAI-1 promoter polymorphism in healthy subjects from the west of Mexico. Association with biochemical and hematological parameters. Ann Genet. 2004;47(2):155-62. doi: 10.1016/j.anngen.2003.12.001.

R20. Roncal C, Orbe J, Rodriguez JA, Belzunce M, Beloqui O, Diez J, Páramo JA. Influence of the 4G/5G PAI-1 genotype on angiotensin II-stimulated human endothelial cells and in patients with hypertension. Cardiovasc Res. 2004;63(1):176-85. doi: 10.1016/j.cardiores.2004.03.023.

R21. Liu SQ, Xue YM, Yang GC, He FY, Zhao XS. relationship between plasminogen activator inhibitor-1 gene 4G/5G polymorphism and type 2 diabetic nephropathy in Chinese Han patients in Guangdong Province. J First Mil Med Univ. 2004; 4(8): 904-906.

R22. Chen YL, Zhang JX, Wang PX, Cui BZ, Zhao FM, Mao YM, Li JY, Bi YY, Li H. Association of4G/5G polymorphism in PAI1 promoter with PAI1 level in deep vein thrombosis. Chin J Med Genet. 2005; 22 (6): 624-627.

Meigs JB, Dupuis J, Liu C, O'Donnell CJ, Fox CS, Kathiresan S, Gabriel SB, Larson MG, Yang Q, R23. Herbert AG, Wilson PW, Feng D, Tofler GH, Cupples LA. PAI-1 Gene 4G/5G polymorphism and risk of type 2 diabetes in a population-based sample. Obesity (Silver Spring). 2006;14(5):753-8. doi: 10.1038/oby.2006.85.

R24. Zietz B, Leonhardt K, Schäffler A. Kandidatengene des Diabetes mellitus Typ 2 Gibt es einen Gen-Dosiseffekt für Risikofaktoren sowie mikro-und makrovaskuläre Folgeerkrankungen? Med Klin (Munich). 2006;101(8):605-16.

R25. Wang L, Liu Y, Guo H. Associations of 4G/5G promoter polymorphism of PAI-1 with PAI-1 antigen content and type 2 diabetic nephropathy. Chinese Journal of Gerontology. 2007; 27: 1485-1487.

R26. Karadeniz M, Erdogan M, Berdeli A, Saygili F, Yilmaz C. 4G/5G polymorphism of PAI-1 gene and Alu-repeat I/D polymorphism of TPA gene in Turkish patients with polycystic ovary syndrome. J Assist Reprod Genet. 2007;24(9):412-8. doi: 10.1007/s10815-007-9160-7.

R27. Corsetti JP, Ryan D, Moss AJ, Rainwater DL, Zareba W, Sparks CE. Plasminogen activator inhibitor-1 polymorphism (4G/5G) predicts recurrence in nonhyperlipidemic postinfarction patients. Arterioscler Thromb Vasc Biol. 2008;28(3):548-54. doi: 10.1161/ATVBAHA.107.155556.

R28. Kucukarabaci B, Gunes HV, Ozdemir G, Cosan D, Ozbabalik D, Dikmen M, Degirmenci I. Investigation of association between plasminogen activator inhibitor type-1 (PAI-1) gene 4G/5G polymorphism frequency and plasma PAI-1 enzyme activity in patients with acute stroke. Genet Test. 2008;12(3):443-51. doi: 10.1089/gte.2008.0025.

R29. Wang H, Madhusudhan T, He T, Hummel B, Schmidt S, Vinnikov IA, Shahzad K, Kashif M, Muller-Krebs S, Schwenger V, Bierhaus A, Rudofsky G, Nawroth PP, Isermann B. Low but sustained coagulation activation ameliorates glucose-induced podocyte apoptosis: protective effect of factor V Leiden in diabetic nephropathy. Blood. 2011;117(19):5231-42. doi: 10.1182/blood-2010-10-314773.

R30. Katrancıoğlu N, Karahan O, Kurtulgan HK, Sanrı US, Kılıç AT. PAI-1 4G/4G gene polymorphism is associated with higher serum lipid level in Turkish population. Cumhuriyet Med J. 2011; 33: 307-311.

R31. Fernandes KS, Sandrim VC. 4G/5G polymorphism modulates PAI-1 circulating levels in obese women. Mol Cell Biochem. 2012;364(1-2):299-301. doi: 10.1007/s11010-012-1230-1.

R32. Sun SK, Qin Y, Tian QW, Tian YF, Zhai MX. Relationship between PAI-1 4G/5G polymorphism and metabolic syndrome in postmenopausal women. Int J Endocrinol Metab. 2013; 33(6): 361-365.

R33. Xu F, Liu H, Sun Y. Association of plasminogen activator inhibitor-1 gene polymorphism and type 2 diabetic nephropathy. Ren Fail. 2016;38(1):157-62. doi: 10.3109/0886022X.2015.1089464.

R34. Karabouta Z, Makedou A, Argiriou A, Xanthopoulou E, Papandreou D, Rousso I, Athanassiadou-Piperopoulou F. 4G polymorphism of plasminogen activator inhibitor-1 (PAI-1), PAI-1 plasma levels, and lipid profiles in overweight/obese children and adolescents. 55 th Annual ESPE Meeting. 2016.

R35. Sahana N, Vivekananda B, Abinaya M. Association of Plasminogen Activator Inhibitor-1 (PAI-1) 4G/5G and Apolipoprotein E polymorphisms with risk of Myocardial Infarction in Indian Tamil Population. Research Journal of Biotechnology. 2018; 13(1): 91-98.

R36. Chen TH, Hsu CM, Hsu HC, Chiu CT, Su MY, Chu YY, Chang ML. Plasminogen activator inhibitor-1 is associated with the metabolism and development of advanced colonic polyps. Transl Res. 2018;200:43-53. doi: 10.1016/j.trsl.2018.05.010.

R37. Borisova EP, Kylbanova ES, Asekritova AS. Polymorphisms of Genes Involved in Endothelial Dysfunction in the Yakuts with COPD and Metabolic Syndrome. International Journal of Biomedicine. 2018; 8(2): 134-138.

T38. Khalaf FA, Ibrahim HR, Bedair HM. Plasminogen activator inhibitor-1 gene polymorphism as a risk factor for vascular complications in type 2 diabetes mellitus. Egyptian Journal of Medical Human Genetics. 2019; 20: 18.

R39. Oh J, An HJ, Kim JO, Jun HH, Kim WR, Kim EJ, Oh D, Kim JW, Kim NK. Association between Five Common Plasminogen Activator Inhibitor-1 (PAI-1) Gene Polymorphisms and Colorectal Cancer Susceptibility. Int J Mol Sci. 2020;21(12):4334. doi: 10.3390/ijms21124334.

R40. Bayramoglu A, Bayramoglu G, Urhan Kucuk M, Guler HI, Arpaci A. Genetic variations of Renin-angiontensin and Fibrinolytic systems and susceptibility to coronary artery disease: a population genetics perspective. Minerva Cardioangiol. 2020. doi: 10.23736/S0026-4725.20.05212-3.
